# Pomegranate Quality from Consumers’ Perspective: Drivers of Liking, Preference Patterns, and the Relation between Sensory and Physico-Chemical Properties

**DOI:** 10.3390/foods13132118

**Published:** 2024-07-02

**Authors:** Ana Pons-Gómez, Bárbara Delpozo, Julián Bartual, Cristina Besada

**Affiliations:** 1 Sensory and Consumer Science Group, Postharvest Department, Valencian Institute for Agricultural Research (IVIA), CV-315, Km. 10.7, 46113 Valencia, Spain; annapons92@gmail.com (A.P.-G.); barbarads.eno@gmail.com (B.D.); 2 Agricultural Experiment Station of Elche (AESE), CV-855, Km. 1, 03290 Alicante, Spain; bartual_jul@gva.es

**Keywords:** acidity, woody flavour, new cultivar, pomegranate, preferences, consumer, drivers of liking

## Abstract

Acquiring information on consumer preferences for the sensory properties of pomegranates is a cue for breeding programmes to set their quality targets and promote the consumption of this particularly healthy fruit. In this study, a total of 12 pomegranate varieties were evaluated, including commercial (Valenciana, Iliana, Tastem, Rugalate, Wonderful, Mollar 49 y Mollar 45) and new varieties (Ref 102, Ref 383, H3/27, and D27/12). For the first time, consumers not only scored their acceptance of different pomegranate varieties but also described their sensory properties using CATA questions. This approach allowed us to identify the main drivers of liking, preference patterns, and the relationship between sensory and physico-chemical properties. Of all the sensory attributes, acidity intensity was revealed as the main driver of liking/disliking, and two different consumer preference patterns were identified: ‘low acid pomegranate lovers’ and ‘acid pomegranate lovers’. Seed properties like the intensity of woody flavour and seed/aril ratio were also key drivers for preferences. A relationship between sensory and physico-chemical properties was also established. Interestingly, sweetness perception correlated more strongly with low titratable acidity levels than with high total soluble solids levels, corroborating that acidity level is a key measurement for quality assessments. ‘D27/12’ was the most promising of the new varieties for having the well-appreciated internal properties of the ‘Mollar’ varieties and external and internal red colouration, which makes it much more appealing to consumers. This study shows that there is still room on the market for pomegranate varieties with very different sensory properties and highlights the need to develop sensory labels that help consumers make the right choices.

## 1. Introduction

In recent years, the market interest in pomegranate (*Punica granatum* L.) has increased significantly because it is now considered a functional food due to its health benefits [1,2,3]. In response to this growing demand, various breeding programmes have been established around the world to produce new superior-quality cultivars [4,5]. For these programmes to be successful, it is essential for breeders to set their objectives based on consumer preferences. However, to do so, information is lacking.

To date, evaluations of the quality of new cultivars have focused on physico-chemical characteristics [6,7,8,9]. However, consumers have the last word because the global perception of eating fruit depends on many interacting factors. For this reason, breeding programmes of fruit like citrus [10,11] or tomato [12,13,14] have included consumer sensory studies as a key step in the selection process.

To the best of our knowledge, only one study that involved consumers has been carried out on pomegranate [15]. In this study, Israeli consumers evaluated the acceptance of 18 new cultivars, while sensory characteristics were described by a trained panel. However, in recent decades, sensory science has significantly evolved, and the focus has completely shifted to consumers. New tests have been developed to allow consumers to describe organoleptic food properties: flash profiling, projective mapping, check-all-that-apply (CATA) questions, etc. [16]. Of them, CATA questions have become one of the most widely used because they provide a rapid method that is easy and convenient for consumers. This methodology has been recently applied to obtain consumer descriptions of the sensory properties of new strawberry and mandarin cultivars [10,11,17].

In this study, the CATA questions test was used to allow consumers to describe the organoleptic characteristics of four new pomegranate cultivars obtained from the breeding programme of the Valencian Institute of Agricultural Research (Valencia, Spain) and Agricultural Experimental Station of Elche (Elche, Spain) (IVIA-AESE programme), compared to eight cultivars available on the market during the corresponding harvest periods. This information, combined with acceptance scores, allowed us to identify consumers’ drivers of liking and their main preference patterns. In addition, a relationship was established between the sensory properties described by consumers and the analytically determined physico-chemical properties.

## 2. Materials and Methods

### 2.1. Samples

Thirty-five fruits from 12 pomegranate cultivars were harvested at the optimum ripening stage from the trees grown in the orchards of the IVIA-AESE programme (Elche, Spain). The two new cultivars, ‘Ref 102’ and ‘Ref 383’, were harvested early in the season (12 September, Harvest 1) and evaluated along with commercial cultivars ‘Acco’, ‘Iliana’, ‘Tastem’, and ‘Valenciana’. Late in the season (13 October, Harvest 2), two new cultivars ‘H3/27’ and ‘D27/12’ and four commercial cultivars ‘Mollar 49’, ‘Mollar 55’ (selected clones of ‘Mollar de Elche’), ‘Rugalate’, and ‘Wonderful’ were evaluated. The new cultivars ‘D27/12’ and ‘H3/27’ were obtained by crossing ‘Mollar’ and ‘Wonderful’. ‘Ref 383’ was obtained by F2 backcrossing with the recipient parent. ‘Ref 102’ is an open pollination seedling.

Further, 15 fruits from each cultivar were used for the physico-chemical analysis, with the other 20 for the sensory evaluation.

### 2.2. Physico-Chemical Evaluation

The 15 pomegranates were divided into three replicates of 5 fruits. After removing arils by hand, a portion was reserved for the characterisation of aril/seed size and firmness. The rest were used to obtain three juices and to determine the following variables: juice colour, total solids (TSS), titratable acid (TA), maturity index (MI), juice yield (JY), and total anthocyanin content (TAC).

To estimate aril size, the maximum width (w) and length (l) were measured on 30 arils with a digital calliper. Then the fleshy part of the arils was removed, and the same measurements were taken on the seeds. From these data, the aril and seed volumes were calculated by assuming a cylindrical shape (V = π·w2·l/4), expressed as mm^3^. Finally, the percentage of the total aril volume that was occupied by seeds was calculated (% seed-aril).

Firmness was determined on 50 arils and seeds per cultivar using an Instron universal testing machine (model 3343, Instron Ltd., Buckinghamshire, UK). The test was measured with a 3-cm plunger. The results are expressed as force/deformation (N/mm).

The three juice samples were obtained with a blender (JU3708, Moulinex, Bagnolet, France). Juice colour was measured by a Minolta colourimeter (model CR-300; Minolta Co. Ltd., Osaka, Japan). The mean values of the CIELab colour parameters were calculated.

Juice yield was expressed as a percentage in relation to the total weight of the arils used to obtain juice. The TSS of juice was determined by a digital refractometer (Atago PR-1, Atago Co., Ltd., Tokyo, Japan) (EN 12143: 1996) [18]. The results are expressed as a percentage. TA was determined by an automatic titrator (HI 901 potentiometric titrator with HI 104 electrode, Hanna Instruments, Gipuzkoa, Spain) with 0.1 N NaOH solution (EN 12147: 1996) [19]. The result was expressed as grams of citric acid per 100 mL of juice. The MI was calculated as TSS/TA.

TAC was determined spectrophotometrically (Multiskan Spectrum, Vantaa, Finland) by the differential pH method [20]. It is expressed as mg/L of cyanidin-3-glucoside.

### 2.3. Consumer Study

A prerequisite for people to participate in this study was to habitually consume fruit (at least once a week). Participation was completely voluntary. In each sensory test (Harvest 1 and Harvest 2), 94 consumers participated. In both cases, consumers evaluated the acceptability of samples and described the main sensory properties using the CATA questions. Consumers’ ages ranged from 18 to 61 years at Harvest 1 and from 19 to 59 years at Harvest 2. The female/male ratio was 65/35 and 62/38 for Harvest 1 and Harvest 2, respectively.

Evaluations were carried out in a standardised test room (ISO 8589: 2007) [21]. Firstly, consumers were shown pictures of two fruits per cultivar (Figure S1). They were asked to indicate how much they liked the external appearance of each cultivar on a 9-point hedonic scale (from 1 = I dislike it very much to 9 = I like it very much).

Then participants were served 15 g of arils in cups coded with three-digit random numbers, along with a spoon. They were first asked to rate their appearance and were then instructed to taste samples and rate their acceptance. In both cases, they used a 9-point hedonic scale. After tasting samples again, they described the main organoleptic properties using the CATA question test. To this end, they were provided with a list of attributes and asked to check all the attributes that they perceived in the sample that they were tasting. The list included 26 attributes related to the taste and texture of arils and seeds. Descriptors were first selected based on previous studies on pomegranates [15,22,23]. Then the list was adapted based on the samples’ specific properties following the procedure described by Tarancón et al. [10]. The attributes included on the final list were the following: not very sweet, sweet, very sweet, not very acid, acid, very acid, no pomegranate flavour, not much pomegranate flavour, medium pomegranate flavour, intense pomegranate flavour, no woody flavour, not much woody flavour, medium woody flavour, intense woody flavour, soft aril, medium aril firmness, firm aril, juiceless, juicy, lots of fleshy parts, seed occupies all, soft seed, hard seed, bitter, astringent, and crunchy.

In all cases, samples were presented monadically, following Williams’ Latin square design. To taste arils, participants were provided with water to cleanse their palates between samples.

Finally, consumers answered some demographic questions about gender and age.

The scientific directorate of the IVIA revised the protocol and procedures of this study and stated a waiver of consent. All the articles from the Declaration of Helsinki and the 2016/679 EU Regulation regarding personal data processing were met.

### 2.4. Statistical Analysis

One-way Analysis of Variance (ANOVA) was used to assess variation in the physico-chemical parameters among cultivars after confirming that they met the requirements of normal distribution and homoscedasticity. Differences among cultivars were determined according to the multiple comparisons of Fisher’s LSD test (*p* ≤ 0.05).

Differences in the mean values of acceptability between cultivars were assessed by the Kruskall–Wallis test and Dunn’s multiple comparison tests (*p* ≤ 0.05).

To investigate consumer groups with similar preference patterns, a hierarchical cluster analysis (HCA) was performed on the acceptance data. Euclidean distances (dissimilarity), Ward’s techniques (agglomeration), and automatic truncation were used.

For the CATA question responses, the frequency of citing attributes in CATA was determined per sample. The non-parametric Cochran’s Q test was performed on the raw binary CATA data to determine the significance between samples for each sensory attribute (*p* ≤ 0.05). A multiple factor analysis (MFA) was performed on the dataset of each harvest by taking into account the statistically significant attributes to differentiate samples and the acceptance scores of the two consumer groups identified in the HCA. Penalty analyses were performed using the CATA data and the acceptance values when tasting arils.

Finally, the MFA and the correlation matrix were performed to investigate the relationship between sensory properties as described by consumers and the instrumentally determined physico-chemical properties.

All the statistical analyses were carried out using the XLSTAT software (version 2023, Addinsoft Inc., New York, NY, USA).

## 3. Results

### 3.1. Physico-Chemical Parameters

**Figure 1:**
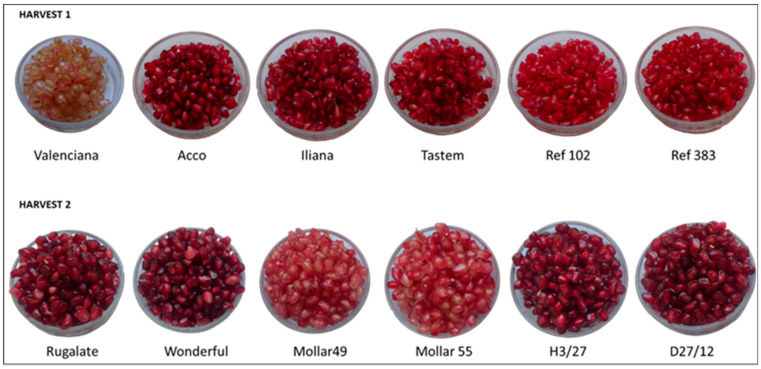
Aspect of arils.

Figure 1 shows the appearance of arils. After evaluating the colour data of the juice, parameters L* and a* were identified as those that better reflected the colour differences in arils as perceived by the naked eye (Table 1). Such differences in colour were mostly explained by TAC (Table 1).

At Harvest 1, two cultivars stood out for their low and high anthocyanin content, respectively. The ‘Valenciana’ arils had the lowest anthocyanin levels (24 mg/L). It was the only variety with withe-pink arils, which was reflected in a mostly white juice colour that showed negative a* values (−0.5) and the highest positive L* values (+29.3). The highest anthocyanin concentration (765 mg/L) was detected in ‘Acco’. This cultivar had the darkest red colour, which was reflected by the lowest L* values of all the cultivars. At Harvest 2, ‘Mollar 55’ and ‘Mollar 49’ differed from the rest by having a less intense red aril colouration (Figure 1), which was associated with a lower total anthocyanin concentration, as reflected by higher L* values (Table 1).

Of the cultivars from Harvest 1, the TSS range of values went from around 15% and determined in ‘Acco’ and ‘Tastem’ to 17.5% in ‘Ref 383’. For TA, cultivars ‘Valenciana’, ‘Iliana’, and ‘Ref 383’ had acidity levels below 0.3 g/100 mL. The cultivar with the highest acidity values, 0.65 g/100 mL, was ‘Acco’. The higher acidity level of ‘Acco’, combined with its low TSS content, made this cultivar stand out from the others for having the lowest MI. The MI of ‘Acco’ was 23, while the other five cultivars had MI values between 33.5 and 67.5. Melgarejo et al. [24] classified pomegranate cultivars according to their MI into acid (MI: 5–7), sweet-acid (MI: 17–28), and sweet (MI: 32–96). Based on this classification, ‘Acco’ would be a sweet-acid cultivar, while ‘Valenciana’, ‘Iliana’, and ‘Tastem’ and the two new cultivars ‘Ref 102’ and ‘Ref 383’ should be classified as sweet cultivars.

The picture was different at Harvest 2. In general, all six evaluated cultivars had higher TSS content and a higher acidity level than the cultivars evaluated at Harvest 1. MIs ranged between 15.7 and 39.9, with the highest values for ‘Mollar 49’ and ‘Mollar 55’, which can be classified as sweet cultivars together with ‘H3/27’. The lowest MIs were for ‘Wonderful’, which stood out for its high acidity level. This cultivar can be classified as acid, while ‘D27/12’ and ‘Rugalate’, with MI values close to 28, can be considered sweet-acid cultivars, according to Melgarejo et al. [24].

For JY, no significant differences were detected among the 12 evaluated cultivars because they all obtained values of around 70–75%, which are in accordance with the values reported in previous studies [25].

One aspect to consider when evaluating the physico-chemical properties of pomegranate is aril size. The characteristics of pomegranate seeds are also an important factor to evaluate because they have been reported to affect consumer acceptance [15]. Two parameters seem to be determinant: one is the ratio seed/aril [7,26] and the other is seed hardness [27]. The ratio seed/aril indicates the percentage of the total volume of arils occupied by seeds.

Aril volume provides an idea of aril size (Table 2). At Harvest 1, cultivars ‘Valenciana’, ‘Tastem’, and ‘Ref 383’ had significantly larger arils than the other cultivars. The aril size of the cultivars evaluated at Harvest 2 was larger than that of the cultivars at Harvest 1 in all cases. Of these, ‘Mollar 49’ and ‘Mollar 55’, and the new cultivar ‘H3/27’, stood out for having the largest arils.

At Harvest 1, the percentage of aril size occupied by seeds was 11.9–13.2% for most cultivars, except for ‘Ref 102’, with values of 15% (Table 2). Values were lower for the cultivars evaluated at Harvest 2. Three cultivars (‘Mollar 55’, ‘Mollar 49’, ‘D27/12’) had values below 8%, while the values of the commercial cultivars ‘Rugalate’ and ‘Wonderful’, and the new cultivar ‘H3/27’, were between 8% and 10%.

There were no differences in seed firmness in the cultivars evaluated at Harvest 1, with values ranging from 24 to 29 N/mm. The picture was very different at Harvest 2 when the firmness values of seeds ranged from 29.6 to 80.1 N/mm. The softest seeds were those of ‘D27/12’ with values close to 30 N/mm and were, therefore, similar to those of the Harvest 1 cultivars. Far from these values came ‘Wonderful’ and ‘H3/27’, which had the hardest seeds with firmness values above 74 N/mm. The seed firmness of cultivars ‘Mollar 55’, ‘Mollar 49’, and ‘Rugalate’ was intermediate (35–45 N/mm).

### 3.2. Sensory Characterisation

#### 3.2.1. External Appearance and Aril Appearance

The first task that consumers were asked to perform in the sensory study was to indicate how much they liked fruit and aril appearance (Figure S1).

*Harvest 1*

At Harvest 1, consumers very much liked the external appearance of ‘Acco’, ‘Ref 102’, ‘Ref 383’, ‘Tastem’, and ‘Iliana’ (Figure S2A). The lesser acceptance of the ‘Valenciana’ appearance must be associated with the presence of yellowish areas on the skin, together with some visible damage.

Regarding aril appearance, all the cultivars scored between 6.4 and 7.3, except for ‘Valenciana’, whose yellow-pink arils were not appealing to consumers (Figure 1 and Figure S3A).

*Harvest 2*

The Harvest 2 results corroborated consumer preferences for pomegranates with red skin rather than yellow-pink skin. Thus, ‘Mollar 49’ and ‘Mollar 55’, whose fruits were mainly yellow, had the lowest scores (Figure S2B). This result agrees with Pons-Gómez et al. [28], who concluded that colour is an external appearance factor with a strong influence on consumer choice and reported that most consumers like bicolour and red pomegranates but reject those that are mostly yellow.

In addition to external colour differences, ‘Mollar 49’ and ‘Mollar 55’ also differed from the other cultivars due to the colour of their arils, which are paler. This allowed us to corroborate consumer preference for intense red arils rather than pink ones, as observed at Harvest 1 (Figure 1 and Figure S3B). It is important to note that the differences in the red aril tones detected at Harvest 2 (Figure 1) were not reflected in the juice colour (Table 1). To make a more accurate colour determination, it would be interesting to directly evaluate colour on arils in future studies.

#### 3.2.2. Preference Patterns when Tasting Arils

**Figure 2:**
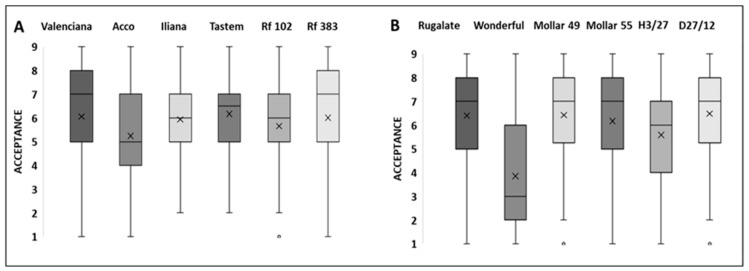
The box-and-wisher plots of the descriptive statistics of the consumer liking scores for the pomegranate cultivars evaluated at Harvest 1 (**A**) and Harvest 2 (**B**). The lower and upper edges of a box represent the first (Q1) and third (Q3) quartiles, while the line and cross within the box show the median and the mean, respectively. The whiskers represent the lower and upper extremes of the data.

Determining the mean liking scores that consumers gave to each cultivar after tasting arils resulted in acceptance values from 5.2 to 6.2 in the cultivars evaluated at Harvest 1 and from 3.9 to 6.5 at Harvest 2 (Figure 2). The box-and-whisker plots indicated wide variability in consumer scores because they spread over the entire hedonic scale for most cultivars. Thus, we performed the HCA on the data for each harvest, which allowed us to identify two different preference patterns for all the consumers who participated in each sensory session (Figure S4).

Once consumers had been grouped according to their preference patterns, multifactor and penalty analyses were performed independently with the data for each sensory test to investigate the way each group described cultivars and identify drivers of liking. To perform them, we used the data from the liking scores and the attributes from the CATA questions that had a significant effect on differentiating samples.

*Harvest 1*

**Figure 3:**
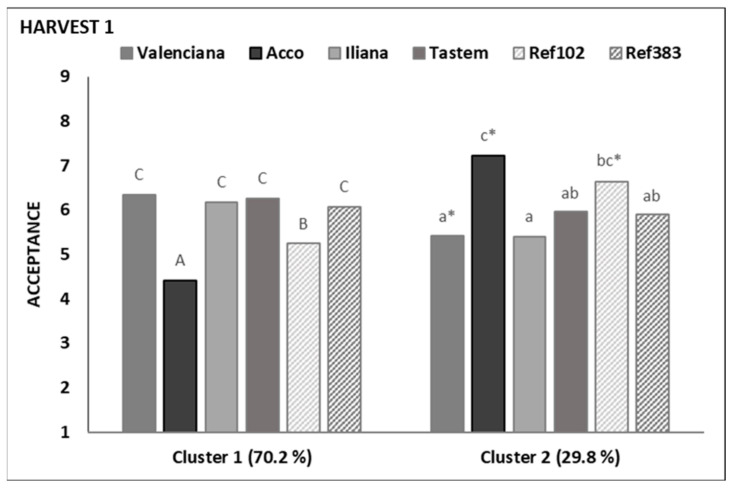
The mean acceptance scores are given by consumers for the cultivars evaluated at Harvest 1. For each cluster, different letters among cultivars indicate significant differences (*p* ≤ 0.05) according to the Kruskal-Wallis test. * denotes significant differences between the scores given to the same cultivar by the consumers in the two clusters.

Of the two preference patterns identified at Harvest 1, Cluster 1 consisted of 66 consumers (70%) and Cluster 2 comprised 28 (30%) (Figure S4A and Figure 3). The main difference among clusters was acceptance of cultivar ‘Acco’, which was rejected by Cluster 1 consumers but was the most liked cultivar for Cluster 2 consumers. Similarly, but not with such a marked effect, it was also observed for ‘Ref 102’. ‘Valenciana’ was the only cultivar that was better scored by the Cluster 1 consumers compared to the Cluster 2 ones. The other three cultivars, ‘Illiana’, ‘Tastem’, and ‘Ref 383’, were scored similarly by both consumer groups.

**Figure 4:**
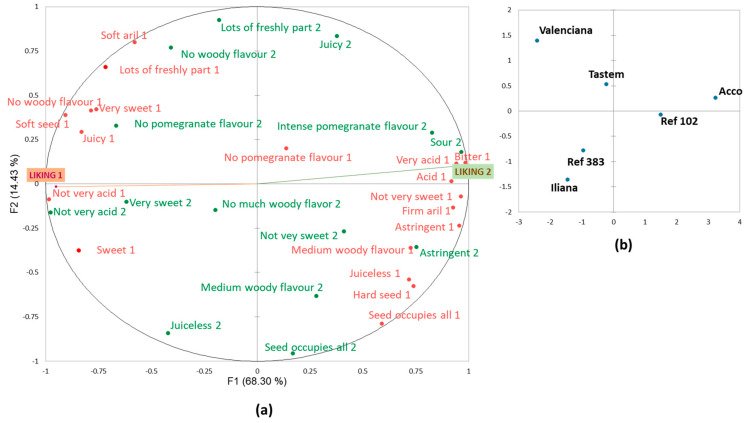
The MFA of the CATA questions at Harvest 1 when taking the responses of Clusters 1 (red font) and 2 (green font) as active datasets and the mean liking scores as supplementary data. (**a**) sensory attributes and liking; (**b**) sample representation.

The MFA (Figure 4) showed the way that each consumer group described samples. The first dimension explained 68% of the variability, while only 14% was explained by the second one. The cultivars described by consumers as being more different from one another were ‘Valenciana’ and ‘Acco’, which were on the opposite ends of the first dimension. The other four cultivars were allocated in the middle along the first dimension. ‘Ref 102’ was closer to ‘Acco’, while ‘Ref 383’, ‘Iliana’, and, to a lesser extent, ‘Tastem’, were closer to ‘Valenciana’.

**Figure 5:**
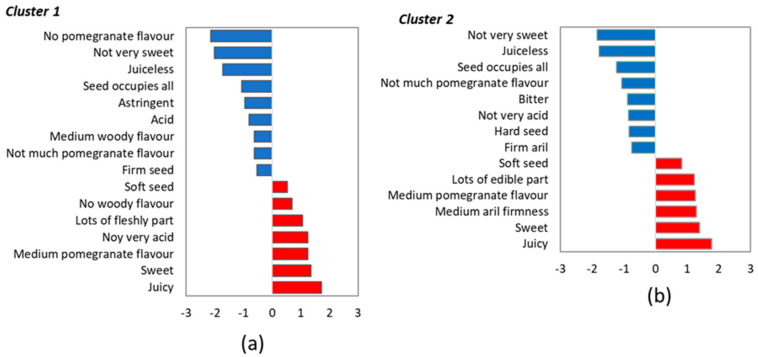
The penalty analysis was applied to the dataset for each cluster identified at Harvest 1: Cluster 1 (**a**), Cluster 2 (**b**). Only the attributes with a significant effect (*p* < 0.05) were included.

It is worth mentioning comparatively the way that the consumers from both clusters described cultivars ‘Acco’ and ‘Ref 102’, which proved to be the two more relevant cultivars to separate consumers by their preference patterns. The Cluster 1 consumers, who did not like these cultivars, used adjectives like ‘acid’, ‘very acid’ and ‘not very sweet’, which indicated that they perceived these two sensory attributes at relatively extreme levels, i.e., the fruit was perceived as very acid with no sweetness. They also used terms like ‘bitter’, ‘astringent’, and ‘medium woody flavour’, and paid much attention to texture attributes like ‘hard seed’ and ‘firm aril’. The penalty analysis helped to understand the link between these properties and the low liking scores that these consumer groups gave to ‘Acco’ and ‘Ref 102’. Thus, for Cluster 1 consumers, attributes like ‘acid’, ‘astringent’, and ‘medium woody flavour’ were identified as drivers for disliking, while ‘no woody flavour’ and ‘not very acid’ were drivers of liking (Figure 5a). Therefore, ‘Acco’ and ‘Ref 102’ did not match the Cluster 1 consumers’ preferences, mainly because of their high acidity level, astringency, and woody flavour.

The Cluster 2 consumers, who showed preferences for these two cultivars, described them as acid and astringent and detected an intense pomegranate flavour. They did not use the ‘very’ adjective for acidity, and ‘not very sweet’ was not frequently applied. For this consumer group, woody flavour was not relevant for differentiating cultivars. In fact, unlike the Cluster 1 consumers, neither woody flavour nor astringency was a disliking driver for the Cluster 2 consumers (Figure 5b). It is important to highlight that ‘not very acid’ was penalised by these consumers. The Cluster 2 preferences for ‘Acco’ and ‘Ref 102’ were explained because they like acid pomegranates with a certain astringency level, which they associate with pomegranate flavour. Accordingly, they gave lower liking scores to the other four cultivars, which they described as not very acid or very sweet, and they did not detect a woody flavour in them. They considered that pomegranate flavour was undetectable in these samples (Figure 4).

*Harvest 2*

**Figure 6:**
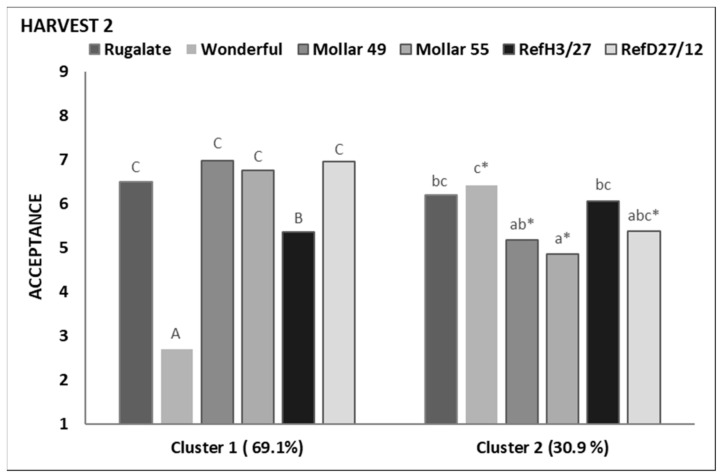
The mean acceptance scores are given by consumers to the cultivars evaluated at Harvest 2. For each cluster, different letters among cultivars indicate significant differences (*p* ≤ 0.05) according to the Kruskal–Wallis test. * denotes significant differences between the scores given to the same cultivar by the consumers of the two clusters.

As with Harvest 1, two preference patterns were identified at Harvest 2 (Figure S4B and Figure 6). Cluster 1 consisted of 65 consumers (69%), with 29 in Cluster 2 (31%). The most significant difference between both consumer groups was the acceptance of the commercial cultivar ‘Wonderful’. The Cluster 2 consumers gave this cultivar a high score, but the Cluster 1 consumers did not like it. The consumers in both clusters also had different acceptances of the cultivars ‘Mollar 49’, ‘Mollar 55’, and ‘Ref D27/12’, which were rated better by Cluster 1 than by Cluster 2. Both clusters shared a liking for ‘Rugalate’.

**Figure 7:**
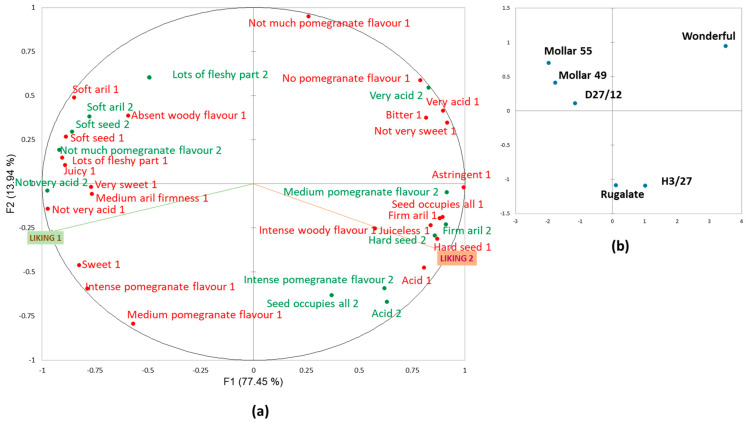
The MFA of the CATA questions at Harvest 2 when taking the responses of Clusters 1 (red font) and 2 (green font) as active datasets and the mean liking scores as supplementary data. (**a**) sensory attributes and liking; (**b**) sample distribution.

According to the description given by the two consumer groups (Figure 7), the first dimension of the MFA (77% of variance) allowed cultivars to be separated into three groups: ‘Mollar 49’, ‘Mollar 55’ and ‘D27/12’ were allocated together to the left in the space, ‘Wonderful’ in the opposite part of the space, and ‘Rugalate’ and ‘H3/27’ in the middle, close to the origin. The second dimension, with a 14% variance, mainly separated ‘Rugalate’ and ‘H3/27’ from the other cultivars.

**Figure 8:**
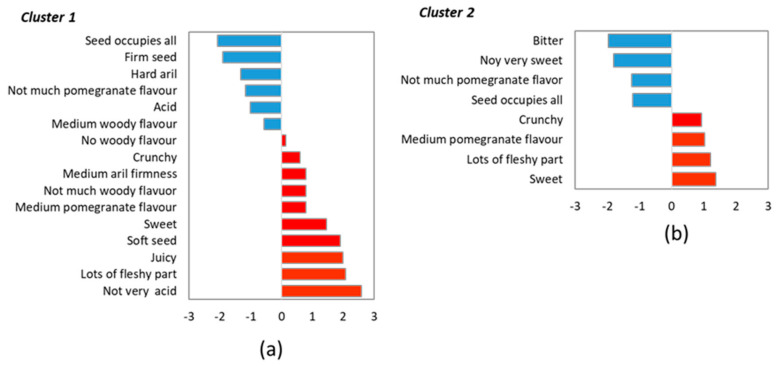
The penalty analysis applied to the dataset for each cluster identified at Harvest 2: Cluster 1 (**a**), Cluster 2 (**b**). Only the attributes with a significant effect (*p* < 0.05) were included.

The Cluster 1 consumers, who rejected ‘Wonderful’, described it as very acid, bitter, not very sweet, and not having much pomegranate flavour. They also used terms like astringent, seed occupied all, hard seed, intense woody flavour, or acid. These last five attributes were also applicable to ‘H23/27’. Most of these attributes were identified as drivers of dislike for the Cluster 1 consumer (Figure 8a). This would explain why they disliked ‘Wonderful’ and gave ‘H3/27’ low scores. Not very acid, lots of fleshy parts, juicy, soft seeds, or not much woody flavour were drivers of liking for these consumer groups, and they used them to define ‘Mollar 55’, ‘Mollar 49’, and ‘D27/12’, which were among their preferred cultivars, together with ‘Rugalate’, with which they shared attributes ‘medium/intense pomegranate flavour’.

Fewer attributes were significant to differentiate cultivars as perceived by Cluster 2 consumers. They defined ‘Wonderful’ as very acid with medium/intense pomegranate flavour and found that seeds were more detectable in both ‘Wonderful’ and ‘H3/27’ than in the other cultivars. Conversely, ‘Mollar 55’, ‘Mollar 49’, and ‘D27/12’ were described as having lots of fleshy parts, soft arils and seeds, not very acid and not much pomegranate flavour. This last attribute was identified as a driver of disliking for this group of consumers (Figure 8b) and is very likely linked with the slightly lower scores given by consumers to these three cultivars compared to ‘Wonderful’ and ‘H3/27’, in which they perceived medium pomegranate flavour.

After taking into account the results of Harvests 1 and 2, acidity was revealed as a key factor for consumer preferences. Thus, at both harvest times, the Cluster 1 consumers showed preferences for the less acid and sweeter cultivars, while the Cluster 2 consumers liked the more acid cultivars the best. In addition, seed and its characteristics were found to be key: the group of consumers who preferred the more acidic cultivars (Cluster 2) did not perceive the presence of seeds and woody taste as negative. In fact, it would seem that the high acidity, woody flavour, and astringency combination was associated with pomegranate flavour. On the contrary, the consumers who preferred cultivars with low acidity rejected not only high acidity levels but also woody flavour and astringency.

One important aspect to mention is the percentage of each consumer type. The ‘low acid pomegranate lovers’ were the majority in both cases and represented around 70% of consumers, while the ‘acid pomegranate lovers’ represented 30%. It is important to mention that most of the consumers who participated in the Harvest 1 sensory session were not the same as those who participated in the session at Harvest 2. Thus, it would seem that these data may represent population preferences.

Puputti et al. [29] investigated the sensitiveness to the main taste qualities of 205 Finnish consumers and reported that 25% of them were hypersensitive, 50% semisensitive, and 25% hyposensitive to acid taste. Sensitiveness may be influenced by genetic background and, therefore, it might not be possible to extrapolate the data obtained from Finland to Spain. However, this study suggests the possibility that sensitiveness to acidity plays a role in the preference pattern herein identified. In fact, when we focus on Harvest 1, the ‘acid pomegranate lovers’ described the more acid cultivars as ‘acid’, while the ‘low acid pomegranate lovers’ used the adjective ‘very’, i.e., ‘very acid’. This may indicate that the ‘low-acid pomegranate lovers’ are more sensitive to acid perception. Undoubtedly, specific studies should be performed to draw a conclusion, but we considered it interesting to mention such a possibility.

In a recent study performed with Spanish consumers, Pons-Gómez et al. [28] highlighted the need to develop sensory labels for pomegranates because most consumers do not know how to make their choices in markets. The need to include sensory labels for fruit has been previously claimed by Fernández-Serrano et al. [30]. The results from the present study on pomegranate, in which two different preference patterns were identified, corroborate the need to provide consumers with sensory labels so they can choose the cultivars that match their preferences. Two parameters were revealed as key to be included on sensory labels, acidity level and sweetness, and seed properties.

#### 3.2.3. Potential of the New Cultivars

When focusing on the potential of the new early-season cultivars, the internal properties of ‘Ref 383’ were similar to those of ‘Iliana’ (Figure 4) and, thus, apparently, it has no advantages over it from this point of view. However, consumers liked the external appearance of ‘Ref 383’ more than that of ‘Iliana’ (Figure S2A), which could imply some commercial advantages.

The ‘Ref 102’ fruit has similar sensory properties to ‘Acco’ but is slightly less acid (Figure 4). Accordingly, the Cluster 1 consumers gave slightly better acceptance scores than they did ‘Acco’ (Figure 3). Hence, we can state that it is a well-accepted cultivar by consumers who like the acid cultivars, and it is not as disgusting as ‘Acco’ for consumers who prefer low-acidity cultivars.

Regarding the potential of the new late-season cultivars, H3/27 had similar external and internal properties to ‘Rugalate’ and does not, therefore, present any advantages from the sensory point of view.

The new cultivar ‘D27/12’ seems more promising. When tasting its fruit, consumers described its sensory properties as being similar to those of ‘Mollar 49’ and ‘Mollar 55’ (Figure 7). It was, therefore, one of those preferred by the majority of consumers (Cluster 1) (Figure 6). The red colouration of ‘D27/12’ is its main advantage compared to both Mollar cultivars because its external and aril appearances were much more appealing to consumers than those of ‘Mollar’ (Figures S2B and S3B).

#### 3.2.4. Relation between Physico-Chemical and Sensory Properties

**Figure 9:**
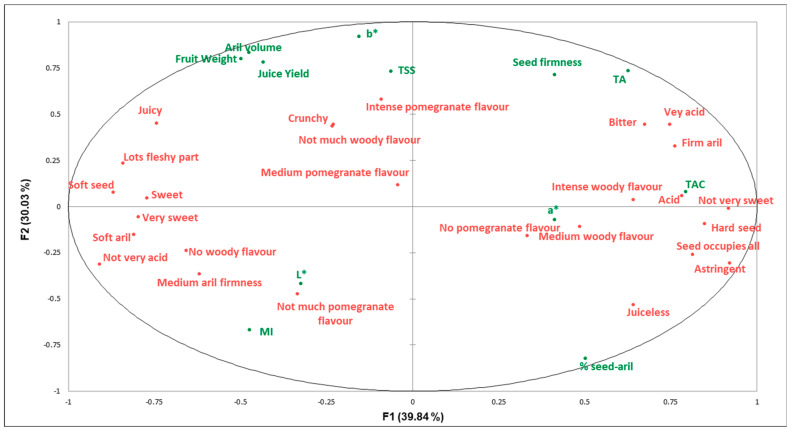
The MFA using the counts of the attributes from CATA questions (red font) and data from the physico-chemical parameters (green font) of the 12 cultivars under study.

A correlation matrix and the MFA were performed with the physico-chemical parameters and sensory attributes to investigate the main correlations among them. Given its size, the correlation matrix appears as Supplementary Material (Figure S5). Here we mention the most relevant results acquired with the MFA (Figure 9), in which closeness in the space between parameters/attributes indicates a positive correlation and the distance in the opposite direction denotes negative correlations.

Some important correlations were observed in the upper and right quarters of Figure 9. Aril size correlated closely to JY, which indicates that the larger the arils are, the juicier they are. Moreover, JY correlated to the ‘juicy’ attribute as detected by consumers. In the opposite lower left quarter was the % seed-aril, which meant that the bigger the aril, the lower the percentage occupied by seeds. In this quarter, and with a negative correlation to JY, the sensory attribute ‘juiciness’ was allocated. The ‘medium woody flavour’ was also in this quarter of the space and showed a certain correlation to the ratio of seed/aril.

In the upper right quarter, a strong correlation was detected between seed firmness and TA. This corroborated the pattern described by Alcaraz-Marmol et al. [31], who found that acid pomegranates have harder seeds than sweet ones.

High acidity levels also correlated to TAC, which indicates that the most acid cultivars were those with intense red arils. There was a clear relation between TA and the acidity level detected by consumers, with the ‘very acid’ attribute being allocated in this quarter. Consumers also related instrumental seed firmness to sensory aril firmness, i.e., the harder the seeds were, the firmer the arils were. Attributes ‘soft aril’ and ‘medium firm aril’ were in the opposite quarter, linked with a negative correlation.

It is important to highlight that sensory attributes ‘sweet’ and ‘very sweet’ were allocated opposite TA rather than close to TSS. This indicates that low acidity levels were necessary for consumers to detect fruit sweetness and to corroborate the importance of acidity as a driver of liking.

It is well-known that the perceived intensity of sweet and acid tastes differs when both tastes are presented in a mixture. The phenomenon whereby the perceived intensity of two tastes in a mixture is less than if they were not mixed, and at the same concentration level, is known as mixture suppression [32]. Research with adults suggests a stronger suppressive effect of acids over sweetness than sugars over acids [33], which helps to explain the key role of pomegranate acidity in consumer preferences. In this study, consumers were classified as ‘acid pomegranate lovers’ and ‘low acid pomegranate lovers’. The latter could also be named ‘sweet pomegranate lovers’. However, we found that low acidity levels were a determinant because otherwise, sweetness might not be perceived, which is why we suggest the acid/low acid classification. This result also highlighted that TA should be considered the key harvesting index rather than TSS or MI.

It is worth mentioning that among the three colour parameters, parameter a* was the one that correlated the best with the total anthocyanin content, even though the correlation was not very strong.

## 4. Conclusions

Our results revealed, for the first time, two preference patterns between pomegranate consumers: ‘low acid pomegranate lover’ (70%) and ‘acid pomegranate lovers’ (30%). This result indicates that there is room on markets for cultivars with very different sensory properties, and highlights the need to use sensory labels that help consumers make the right choices. In addition to the acidity/sweetness level, this label should also include information about seed characteristics. Quantifying the extent to which consumer satisfaction is enhanced by a well-designed sensory label that assists them in their choice may encourage the industry to implement it, and our group is already working on this.

Of the correlations established between the instrumental measurements and sensory characteristics, it is worth highlighting that the sensory perception of high sweetness levels correlated strongly to low TA levels rather than to high TSS concentrations. Therefore, TA should be considered the main harvest index rather than TSS or the MI.

‘D27/12’ is the most promising of the newly evaluated cultivars. The sensory characteristics perceived by consumers when tasting its fruit are very similar to those of the ‘Mollar’ cultivars. However, its main advantage is that it has an external and internal red colour, which makes it much more appealing to consumers. Future studies should determine the postharvest response of this variety. Since it is a late-season variety, it is particularly important to investigate its suitability for cold storage to extend the commercial season with high-quality pomegranates.

## Figures and Tables

**Table 1:** Physico-chemical parameters of the 12 pomegranate cultivars. For each harvest, different letters in the same column represent significant differences according to the LSD test (*p* ≤ 0.05).

Hv.	Cultivars	Juice Colour L*	Juice Colour a*	Juice Colour b*	TAC ^1^ (mg/L)	TSS ^2^ (%)	TA ^3^ (g Citric ac./100 mL)	MI ^4^ (TSS/TA)	JY ^5^ (% Juice)
1	Valenciana	29.3 c	−0.5 a	2.6 a	33.9 a	16.0 b	0.24 a	67.5 c	71.5 a
	Acco	21.4 a	4.7 b	3.0 ab	765.3 e	14.9 a	0.65 d	23.0 a	68.5 a
	Iliana	22.9 b	5.4 b	3.0 ab	462.5 c	16.7 c	0.25 a	66.6 c	72.6 a
	Tastem	22.6 b	5.4 b	3.4 b	572.7 d	15.2 a	0.44 b	34.3 b	73.5 a
	Ref 102	23.5 b	7.8 d	3.5 b	289.7 b	16.4 bc	0.49 c	33.5 b	70.6 a
	Ref 383	23.6 b	6.4 c	4.4 c	409.4 c	17.5 d	0.28 a	63.5 c	69.8 a
2	Rugalate	21.8 a	4.5 a	6.5 b	491.9 c	17.7 ab	0.63 a	27.9 bc	76.1 a
	Wonderful	21.6 a	4.5 a	6.2 ab	605.1 d	18.7 c	1.30 b	15.7 a	74.3 a
	Mollar 49	23.7 b	4.3 a	5.4 ab	98.9 a	17.8 b	0.52 a	34.1 cd	73.9 a
	Mollar 55	25.4 b	4.4 a	4.8 a	123.8 a	18.0 b	0.46 a	39.9 d	77.3 a
	H3/27	21.5 a	4.6 a	6.4 ab	582.9 d	18.9 c	0.63 a	29.7 bc	73.6 a
	D27/12	21.4 a	4.5 a	6.2 ab	435.1 b	17.1 a	0.64 a	26.7 b	76.2 a

TAC ^1^: total anthocyanin content; TSS ^2^: total soluble solids; TA ^3^: titratable acidity; MI ^4^: maturity index; JY ^5^: juice yield.

**Table 2:** Seed characteristics of the distinct pomegranate cultivars. Different letters in the same column indicate significant differences according to the LSD test (*p*-value ≤ 0.05).

Harvest	Cultivars	Aril Size (mm^3^)	% Seed-Aril	Seed Firmness (N/mm)
1	Valenciana	387.1 b	12.2 a	27.5 a
	Acco	305.6 a	13.2 a	24.3 a
	Iliana	326.5 a	12.5 a	28.5 a
	Tastem	391.5 b	12.1 a	29.1 a
	Ref 102	291.0 a	15.0 b	26.8 a
	Ref 383	391.7 b	11.9 a	27.0 a
2	Rugalate	525.0 b	8.2 b	45.2 c
	Wonderful	457.3 a	10.0 c	80.1 d
	Mollar 49	609.4 c	7.6 ab	38.9 bc
	Mollar 55	613.7 c	7.8 ab	34.9 ab
	H3/27	587.0 bc	10.2 c	74.1 d
	D27/12	528.3 b	6.8 a	29.6 a

## Data Availability

The original contributions presented in the study are included in the article/Supplementary Materials, further inquiries can be directed to the corresponding author.

## Supplementary Materials

The following supporting information can be downloaded at: https://www.mdpi.com/article/10.3390/foods13132118/s1, Figure S1: External appearance of the evaluated cultivars; Figure S2: Acceptance of external appearance; Figure S3: Acceptance of aril appearance; Figure S4: Clusters of consumers; Figure S5: Matrix of correlation among sensory and physicochemical data.
