# Correction: Pons-Gómez et al. Pomegranate Quality from Consumers’ Perspective: Drivers of Liking, Preference Patterns, and the Relation between Sensory and Physico-Chemical Properties. *Foods* 2024*, 13,* 2118

**DOI:** 10.3390/foods14020220

**Published:** 2025-01-13

**Authors:** Ana Pons-Gómez, Bárbara Delpozo, Julián Bartual, Cristina Besada

**Affiliations:** 1 Sensory and Consumer Science Group, Postharvest Department, Valencian Institute for Agricultural Research (IVIA), CV-315, Km. 10.7, 46113 Valencia, Spain; 2 Agricultural Experiment Station of Elche (AESE), CV-855, Km. 1, 03290 Alicante, Spain; bartual_jul@gva.es

## 1. Text Correction

In the original publication [1] there was a typo in the Abstract, ‘Illina’ should have been written as ‘Iliana’. Additionally, in both the Abstract and the Conclusions sections, in the sentences “‘H3/27’ was the most promising of the new varieties …” and “‘H3/27’ is the most promising of the newly evaluated cultivars”, the variety ‘H3/27’ should be replaced by ‘D27/12’. The sentences were modified to the correct forms:

‘D27/12’ was the most promising of the new varieties for having the well-appreciated internal properties of the ‘Mollar’ varieties and external and internal red colouration, which makes it much more appealing to consumers.

‘D27/12’ is the most promising of the newly evaluated cultivars.

Additionally, in Section 3.2.2, *Preference Patterns when Tasting Arils*, in the first paragraph of the *Harvest 2* subsection, the sentence contained an error regarding the cluster descriptions. The clusters were mistakenly swapped. The corrected sentence reads:

“The Cluster 2 consumers gave this cultivar a high score, but the Cluster 1 consumers did not like it”.

Similarly, in the same section, in the second paragraph after Figure 8, there was another confusion between Cluster 1 and Cluster 2. The corrected sentence is:

“In addition, seed and its characteristics were found to be key: the group of consumers who preferred the more acidic cultivars (Cluster 2) did not perceive the presence of seeds and woody taste as negative”.

## 2. Error in Figure

In the original publication [1], there was a mistake in Figure S3 (Supplementary Materials). The figure was missing and Figure S2 was duplicated. The corrected Figure S3 appears below. 

The authors state that the scientific conclusions are unaffected. This correction was approved by the Academic Editor. The original publication has also been updated.

## Figures and Tables

**Figure S3:**
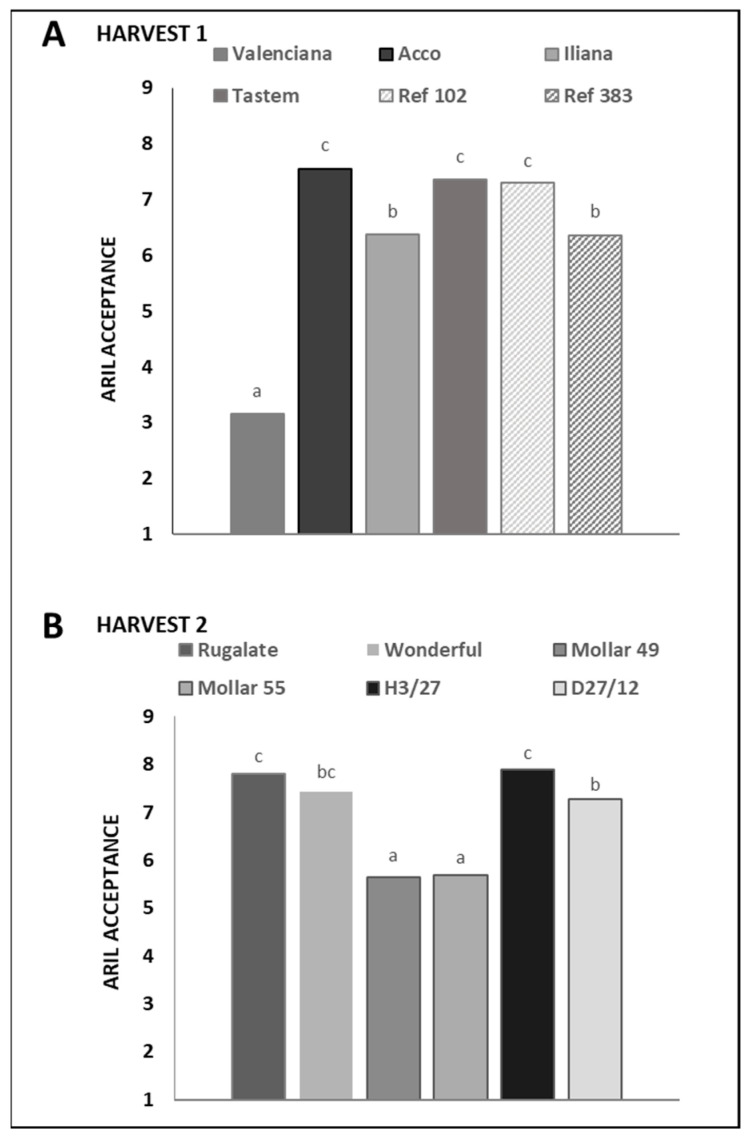
Acceptance of aril appearance. At each harvest, different letters among varieties indicate significant differences (*p* ≤ 0.05) according to Kruskal–Wallis test.
