# Supplementary material for: Pomegranate Quality from Consumers’ Perspective: Drivers of Liking, Preference Patterns, and the Relation between Sensory and Physico-Chemical Properties
Source: Foods. 2024 Jul 2;13(13):2118. doi: 10.3390/foods13132118 (PMC11241000; doi:10.3390/foods13132118)
Supplement: Supplementary file 1 [file foods-13-02118-s001.zip › foods-3054244-supplementary.pdf]

Supplementary material

Tittle: Pomegranate quality from consumers' perspective: drivers of liking, preference patterns, and the relation between sensory and physico-chemical properties

Ana Pons-Gómez [1], Bárbara Delpozo [1], Julián Bartual[2], and Cristina Besada [1],*

[1] Sensory and Consumer Science Group, Postharvest Department, Valencian Institute for Agricultural Research, CV-315, Km. 10.7, 46113 Valencia, Spain

[2] Agricultural Experiment Station of Elche, CV-855, Km. 1, 03290 Alicante, Spain

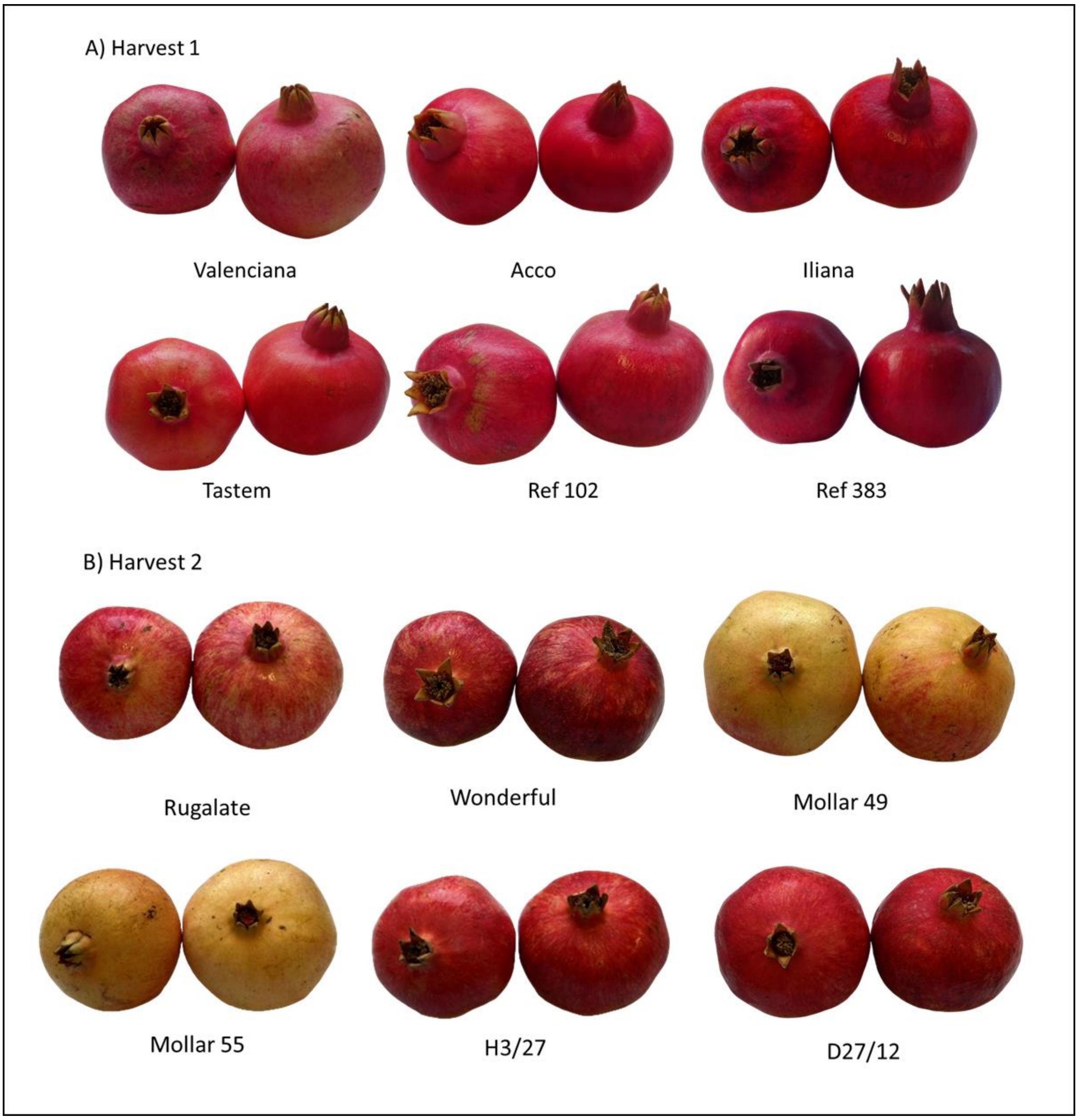

Figure S1. External appearance of the evaluated cultivars.

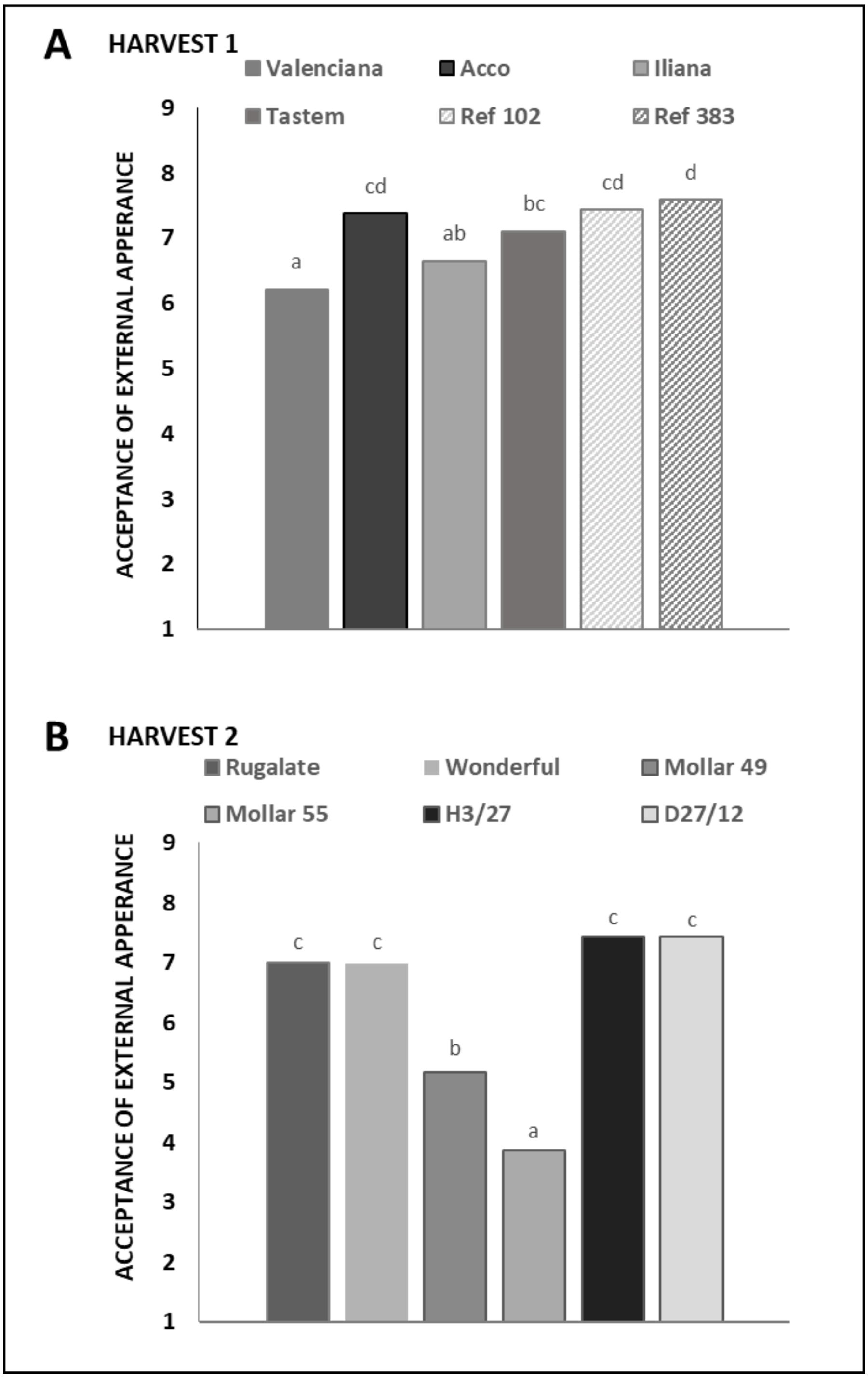

Figure S2. Acceptance of external appearance. At each harvest, different letters among varieties indicates significant differences ($p \leq 0.05$) according to Kruskal–Wallis test

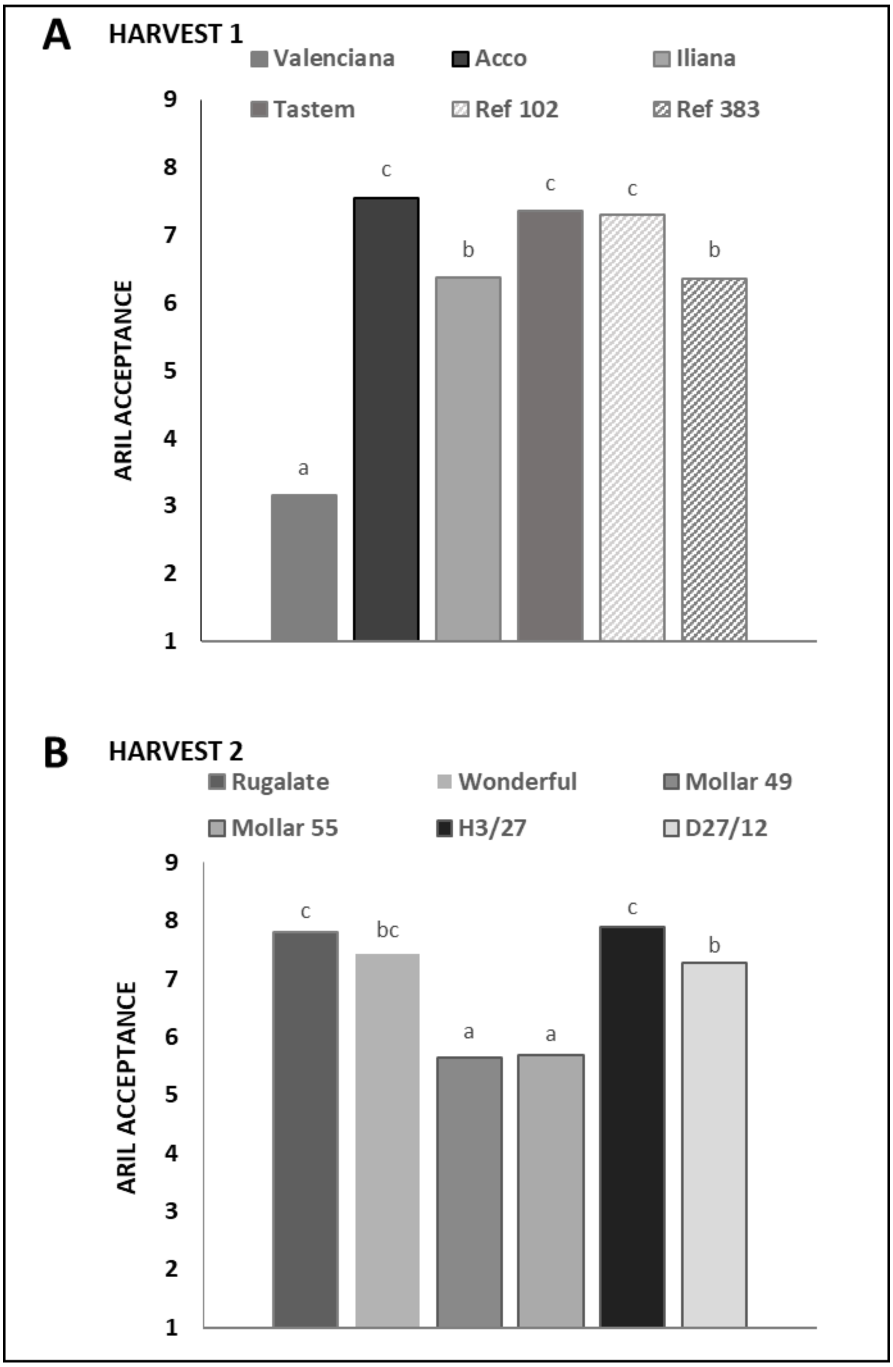

Figure S3. Acceptance of aril appearance. At each harvest, different letters among varieties indicate significant differences ($p \leq 0.05$) according to Kruskal–Wallis test

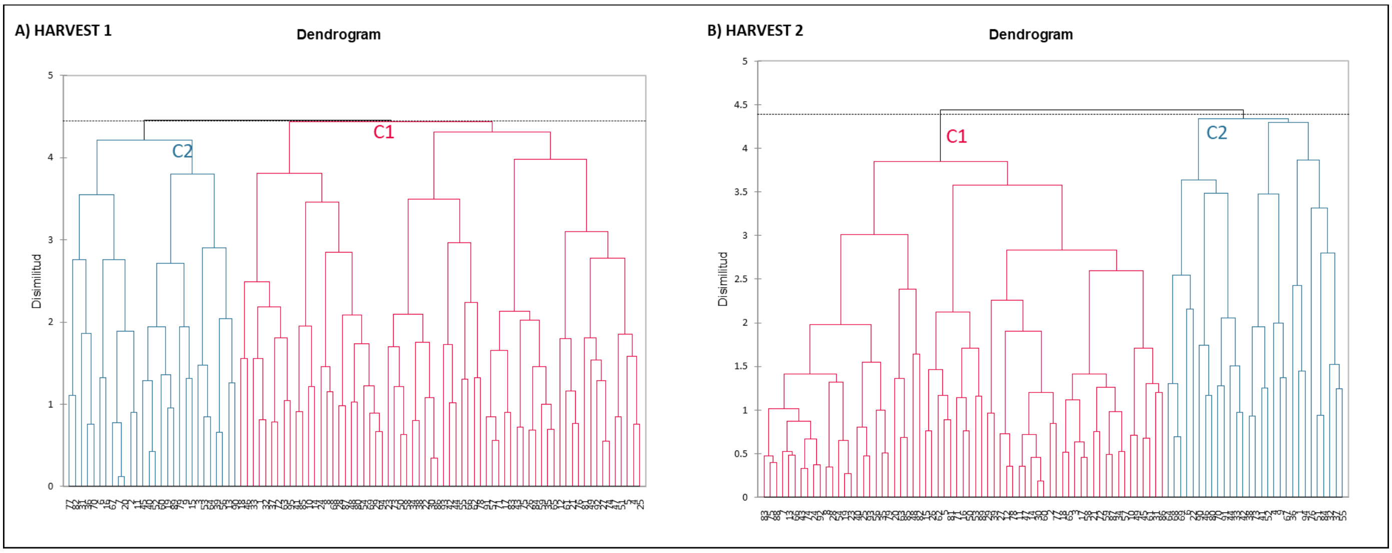

Figure S4. Clusters of consumers identified based on their preferences when tasting the different pomegranate varieties.

	Fruit Weigh	Juice Yield	TSS	TA	MI	TAC	Aril volume	% seed-aril	Seed firmness	Juice colour L*	Juice colour a*	Juice colour b*	Bitter	Astringent	Crunchy	Noy very sweet	Sweet	Very sweet	Not very acid	Acid	Vey acid
Fruit Weigh	1																				
Juice Yield	0.849	1																			
TSS	0.491	0.548	1																		
TA	0.368	0.268	0.422	1																	
MI	-0.444	-0.304	-0.194	-0.817	1																
TAC	-0.336	-0.223	-0.131	0.468	-0.483	1															
Aril volume	0.862	0.783	0.719	0.228	-0.306	-0.296	1														
% seed-aril	-0.919	-0.837	-0.588	-0.281	0.320	0.216	-0.901	1													
Seed firmness	0.320	0.352	0.757	0.750	-0.494	0.297	0.468	-0.310	1												
Juice colour L*	-0.219	-0.313	-0.070	-0.450	0.578	-0.496	-0.096	0.291	-0.036	1											
Juice colour a*	-0.341	-0.132	0.043	0.085	-0.255	0.375	-0.220	0.233	-0.077	-0.632	1										
Juice colour b*	0.801	0.791	0.735	0.552	-0.532	-0.033	0.782	-0.862	0.491	-0.506	0.099	1									
Bitter	0.138	0.039	0.020	0.789	-0.826	0.668	-0.016	-0.056	0.380	-0.652	0.308	0.271	1								
Astringent	-0.662	-0.650	-0.334	0.379	-0.251	0.740	-0.730	0.680	0.101	-0.251	0.361	-0.380	0.532	1							
Crispy	0.276	0.405	0.664	0.179	-0.220	0.121	0.620	-0.372	0.685	0.073	0.049	0.360	-0.077	-0.284	1						
Noy very sweet	-0.351	-0.393	-0.257	0.644	-0.516	0.715	-0.534	0.443	0.243	-0.420	0.308	-0.182	0.793	0.884	-0.270	1					
Sweet	0.301	0.535	0.175	-0.553	0.315	-0.383	0.443	-0.444	-0.273	0.076	0.015	0.278	-0.556	-0.754	0.265	-0.838	1				
Very sweet	0.285	0.119	0.256	-0.523	0.549	-0.814	0.448	-0.295	-0.132	0.628	-0.534	0.024	-0.770	-0.723	0.185	-0.833	0.395	1			
Not very acid	0.105	0.138	-0.063	-0.850	0.707	-0.685	0.251	-0.215	-0.546	0.411	-0.254	-0.144	-0.778	-0.780	0.005	-0.884	0.737	0.739	1		
Acid	-0.261	-0.250	-0.198	0.515	-0.685	0.745	-0.346	0.354	0.238	-0.405	0.441	-0.026	0.645	0.750	0.037	0.714	-0.445	-0.750	-0.813	1	
Vey acid	0.051	-0.008	0.259	0.891	-0.530	0.447	-0.105	0.042	0.649	-0.302	0.031	0.245	0.673	0.590	-0.038	0.780	-0.774	-0.527	-0.878	0.435	1
No pomegranate flavour	-0.217	-0.345	-0.154	0.292	0.144	-0.016	-0.472	0.343	0.039	0.147	-0.324	-0.246	0.217	0.449	-0.627	0.503	-0.677	-0.160	-0.376	-0.043	0.617
Not much pomegranate flavour	-0.202	-0.253	-0.364	-0.451	0.568	-0.419	-0.303	0.252	-0.502	0.149	-0.159	-0.498	-0.240	-0.122	-0.451	-0.044	-0.162	0.239	0.422	-0.573	-0.181
Medium pomegranate flavour	-0.012	0.198	0.164	-0.006	-0.236	0.231	0.182	-0.088	0.206	0.022	0.320	0.193	-0.144	-0.101	0.560	-0.212	0.478	-0.128	-0.025	0.346	-0.245
Intense pomegranate flavour	0.475	0.427	0.410	0.266	-0.569	0.266	0.633	-0.548	0.339	-0.353	0.155	0.633	0.238	-0.184	0.541	-0.207	0.332	0.012	-0.111	0.351	-0.116
Absent woody flavour	0.143	0.022	-0.049	-0.480	0.594	-0.811	0.110	0.016	-0.227	0.703	-0.703	-0.259	-0.627	-0.506	-0.180	-0.558	0.146	0.791	0.562	-0.662	-0.325
Not much woody flavour	0.501	0.654	0.085	0.235	-0.272	0.153	0.283	-0.559	0.087	-0.415	-0.035	0.435	0.150	-0.274	0.161	-0.022	0.322	-0.289	-0.010	-0.034	0.042
Medium woody flavour	-0.358	-0.331	-0.209	0.217	-0.355	0.566	-0.383	0.224	-0.198	-0.624	0.773	0.085	0.428	0.575	-0.280	0.483	-0.175	-0.634	-0.427	0.590	0.175
Intense woody flavour	-0.378	-0.367	0.247	0.324	-0.316	0.577	-0.044	0.325	0.548	-0.020	0.344	-0.132	0.423	0.502	0.484	0.426	-0.405	-0.305	-0.466	0.492	0.316
Soft aril	0.343	0.128	-0.201	-0.511	0.390	-0.824	0.245	-0.260	-0.544	0.268	-0.362	-0.065	-0.489	-0.687	-0.299	-0.583	0.287	0.690	0.716	-0.734	-0.505
Medium aril firmness	-0.007	0.132	-0.497	-0.630	0.370	-0.328	-0.181	-0.035	-0.826	-0.130	0.116	-0.095	-0.459	-0.340	-0.446	-0.434	0.607	0.115	0.553	-0.249	-0.650
Hard aril	-0.163	-0.151	0.433	0.674	-0.440	0.624	0.002	0.151	0.823	-0.044	0.099	0.096	0.549	0.565	0.447	0.577	-0.549	-0.415	-0.723	0.527	0.685
Juiceless	-0.832	-0.734	-0.134	-0.026	0.201	0.447	-0.698	0.736	-0.031	-0.031	0.487	-0.498	0.176	0.748	-0.178	0.527	-0.459	-0.421	-0.328	0.345	0.224
Juicy	0.832	0.734	0.134	0.026	-0.201	-0.447	0.698	-0.736	0.031	0.031	-0.487	0.498	-0.176	-0.748	0.178	-0.527	0.459	0.421	0.328	-0.345	-0.224
Lots fleshy part	0.713	0.584	-0.059	-0.235	0.080	-0.658	0.494	-0.617	-0.323	0.101	-0.514	0.330	-0.347	-0.787	-0.167	-0.618	0.509	0.523	0.544	-0.542	-0.396
Seed occupies all	-0.713	-0.584	0.059	0.235	-0.080	0.658	-0.494	0.617	0.323	-0.101	0.514	-0.330	0.347	0.787	0.167	0.618	-0.509	-0.523	-0.544	0.542	0.396
Soft seed	0.565	0.481	-0.112	-0.418	0.238	-0.703	0.368	-0.517	-0.551	-0.033	-0.285	0.268	-0.397	-0.755	-0.318	-0.650	0.573	0.512	0.670	-0.606	-0.538
Hard seed	-0.565	-0.481	0.112	0.418	-0.238	0.703	-0.368	0.517	0.551	0.033	0.285	-0.268	0.397	0.755	0.318	0.650	-0.573	-0.512	-0.670	0.606	0.538

	No pomegranate flavour	Not much pomegranate flavour	Medium pomegranate flavour	Intense pomegranate flavour	No woody flavour	Not much woody flavour	Medium woody flavour	Intense woody flavour	Soft aril	Medium aril firmness	Hard aril	Juiceless	Juicy	Lots fleshy part	Seed occupies all	Soft seed	Hard seed
Fruit Weigh																	
Juice Yield																	
TSS																	
TA																	
MI																	
TAC																	
Aril volume																	
% seed-aril																	
Seed firmness																	
Juice colour L*																	
Juice colour a*																	
Juice colour b*																	
Bitter																	
Astringent																	
Crispy																	
Noy very sweet																	
Sweet																	
Very sweet																	
Not very acid																	
Acid																	
Vey acid																	
No pomegranate flavour	1																
Not much pomegranate flavour	0.385	1															
Medium pomegranate flavour	-0.722	-0.749	1														
Intense pomegranate flavour	-0.650	-0.755	0.501	1													
Absent woody flavour	0.274	0.475	-0.443	-0.350	1												
Not much woody flavour	-0.239	-0.097	0.222	0.105	-0.356	1											
Medium woody flavour	-0.088	-0.334	0.269	0.190	-0.791	-0.020	1										
Intense woody flavour	-0.111	-0.326	0.209	0.271	-0.409	-0.465	0.204	1									
Soft aril	-0.044	0.596	-0.370	-0.264	0.665	-0.053	-0.406	-0.626	1								
Medium aril firmness	-0.281	0.208	0.118	-0.108	0.118	0.305	0.173	-0.765	0.466	1							
Hard aril	0.211	-0.432	0.101	0.202	-0.405	-0.180	0.083	0.821	-0.803	-0.901	1						
Juiceless	0.337	0.153	-0.151	-0.351	-0.335	-0.431	0.439	0.542	-0.544	-0.291	0.462	1					
Juicy	-0.337	-0.153	0.151	0.351	0.335	0.431	-0.439	-0.542	0.544	0.291	-0.462	-1.000	1				
Lots fleshy part	-0.162	0.139	-0.089	0.092	0.514	0.371	-0.426	-0.809	0.765	0.550	-0.744	-0.898	0.898	1			
Seed occupies all	0.162	-0.139	0.089	-0.092	-0.514	-0.371	0.426	0.809	-0.765	-0.550	0.744	0.898	-0.898	-1.000	1		
Soft seed	-0.163	0.325	-0.222	0.017	0.491	0.285	-0.279	-0.850	0.819	0.713	-0.881	-0.694	0.694	0.918	-0.918	1	
Hard seed	0.163	-0.325	0.222	-0.017	-0.491	-0.285	0.279	0.850	-0.819	-0.713	0.881	0.694	-0.694	-0.918	0.918	-1.000	1

Figure S5. Matrix of correlation among sensory and physicochemical data.
